# Development of clinical guidelines for service provision of functional electrical stimulation to support walking: mixed method exploration of stakeholder views

**DOI:** 10.1186/s12883-021-02299-1

**Published:** 2021-07-05

**Authors:** C Bulley, C Meagher, T Street, A Adonis, C Peace, C Singleton, J. Burridge

**Affiliations:** 1 Queen Margaret University, Edinburgh, UK; 2 University of Southampton, Southampton, UK; 3 Salisbury NHS Foundation Trust, Salisbury, UK; 4 Imperial College London, London, UK; 5 Birmingham Community Healthcare NHS Trust, Birmingham, UK

**Keywords:** Functional electrical stimulation, Upper motor neuron lesion, Multiple sclerosis, Stroke, Spinal cord injury, Drop foot, Lower limb, Paralysis, Clinical guidelines

## Abstract

**Background:**

Over the past 20 years Functional Electrical Stimulation (FES) has grown in clinical use to support walking in people with lower limb weakness or paralysis due to upper motor neuron lesions. Despite growing consensus regarding its benefits, provision across the UK and internationally is variable. This study aimed to explore stakeholder views relating to the value of a clinical guideline focusing on service provision of FES to support walking, how people might use it and what should be included.

**Methods:**

A mixed methods exploration sought the views of key stakeholders. A pragmatic online survey (*n* = 223) focusing on the study aim was developed and distributed to the email distribution list of the UK Association for Chartered Physiotherapists Interested in Neurology (ACPIN). In parallel, a qualitative service evaluation and patient public involvement consultation was conducted. Two group, and seven individual interviews were conducted with: FES-users (*n* = 6), their family and carers (*n* = 3), physiotherapists (*n* = 4), service providers/developers (*n* = 2), researchers (*n* = 1) and distributors of FES (*n* = 1). Descriptive analysis of quantitative data and framework analysis of qualitative data were conducted.

**Results:**

Support for clinical guideline development was clear in the qualitative interviews and the survey results. Survey respondents most strongly endorsed possible uses of the clinical guideline as ensuring best practice and supporting people seeking access to a FES service. Data analysis and synthesis provided clear areas for inclusion in the clinical guidelines, including current research evidence and consensus relating to who is most likely to benefit and optimal service provision as well as pathways to access this. Specific areas for further investigation were summarised for inclusion in the first stage of a Delphi consensus study.

**Conclusions:**

Key stakeholders believe in the value of a clinical guideline that focuses on the different stages of service provision for FES to support walking. A Delphi consensus study is being planned based on the findings.

**Supplementary Information:**

The online version contains supplementary material available at 10.1186/s12883-021-02299-1.

## Background

Lower limb paralysis or weakness leading to ‘foot drop’ is a common gait disturbance in people with upper motor neuron conditions such as Multiple Sclerosis (MS) and stroke [1, 2]. Foot drop is commonly characterised by a difficulty lifting the toes (dorsiflex) when walking due to fatigue, muscle weakness, and/or spasticity [3]. This makes walking very tiring and difficult, with greater likelihood of trips, loss of balance, and falls. Taking part in activities and roles that require walking can be inhibited by fear and loss of confidence, increasing likelihood of social isolation and reduced independence, profoundly affecting quality of life [4]. Foot drop is estimated to affect approximately 20-30% of people with stroke [5].

To address this issue, people often need some form of assistive device, the most commonly prescribed being an ankle foot orthosis (AFO) [6]. In the last twenty years Functional Electrical Stimulation (FES) has been developed and become more commonly used to support walking following an upper motor neuron lesion.

FES works through passing a small amount of electrical stimulation through the nerves of the lower limb which activate the muscles, allowing people with weakness or paralysis to walk more effectively [7]. There is evidence for the positive impacts of FES which has resulted in its recognition as an evidence-based treatment in the Royal College of Physicians [8] Stroke Guidelines and by the National Institute of Clinical Excellence [9]. Following the first Randomised Control Trial (published in 1997) training courses for physiotherapists began [10] and since then FES has moved from the research environment to being a widely used clinical tool. More than 400 UK physiotherapists are now trained to use FES and 8,000-10,000 UK patients in the UK use it. While there is more evidence relating to use of FES to support walking in people following stroke and MS, there is increasing application for people with other types of condition, such as Cerebral Palsy, demonstrated in systematic reviews and meta-analyses [2, 11, 12].

Despite recognition of the effectiveness of FES by professional bodies and a growing evidence base, translation into the development of FES services has been slow [1, 13]. This may be influenced by lack of specific guidance on FES service provision. Most physiotherapists learn to use FES following qualification and professional registration. An online survey by Auchstaetter et al. [14] found that barriers to FES use among Canadian physical therapists includedlack of funding, resources and confidence.

The variable provision of FES services across the UK has substantial financial implications as well as personal, as it is a strategy to reduce risk of falls. Given that falling has a large impact on quality of life for patients and economic burden on the National Health Service costing more than 2.3 billion per year [15], a service evaluation and patient public involvement (PPI) consultation is required of available services that may reduce the cost of falling and improve quality of life. Patient groups representing service users and the Association of Chartered Physiotherapists in Neurology (ACPIN) have called for improvement in FES service delivery. One route for doing this is to develop clinical guidelines, which aim to improve quality and equity of clinical decision-making, thereby supporting better health outcomes [16]. This has led to the development of a project aiming to develop clinical guidelines relating to service provision of FES to support walking. PPI consultation was conducted through using interviews and focus groups to evaluate a current service, while giving valuable insights into the need for clinical guidelines and potential content.

Therefore, this study aimed to explore the views of key stakeholders, with the following objectives:

1. To explore the value of and need for clinical guidelines that focus on service provision of FES to support walking, including how they might be used, and
2. To explore views on ideal content of such clinical guidelines

## Method

A mixed methods exploration sought the views of FES-users, their family and carers, physiotherapists, service providers, developers, researchers and distributors of FES. A pragmatic online survey focusing on the study aim was developed and distributed to the email distribution list of ACPIN. In parallel, a qualitative service evaluation and PPI consultation was conducted with questions relevant to the study aims. Two groups, and seven individual interviews were conducted with the different stakeholders. Descriptive analysis of quantitative data and Framework analysis of qualitative data were conducted. Ethical review of both sub-studies was conducted.

### Online survey

A brief, anonymous online survey was developed for construction in the ‘Survey Monkey’ software system. An invitation was sent by email to the ACPIN membership and conference trade distribution lists with a short explanation (estimated population of around 3000). Inclusion criteria were contained in the study invitation email: aged 18+y, able to respond to a survey in English and involved in the development, research or provision of FES to support walking. The first page contained full information on the study’s purpose, anonymity, inclusion criteria and data management, with advice that they could withdraw at any time by closing their web browser, and that no information would be saved. Once they clicked submit data could not be extracted due to its anonymous nature. If people were willing to consent to participate, they continued onto the next page of the survey. The survey was open for three weeks and automated survey reminders were emailed after one and two weeks.

The survey was developed by the ACPIN FES working group and used primarily closed questions with response options (e.g. Likert scales) (Supplementary 1). A final question was open, to enable elaboration of earlier responses. The survey was piloted online to ensure it worked and establish the completion time of an average of ten minutes.

Data were exported from Survey Monkey into Excel and securely stored in a password-protected server space for use by the research team. Cleaning of the data was conducted to remove responses where no data had been submitted, and where people only responded to questions about their characteristics and none of the questions relevant to the study aims. Descriptive analysis was conducted using frequencies, percentage frequencies and categorisation of responses to open questions.

### Qualitative data collection

A post-positivist approach was used when planning four focus groups: one with FES users, one with family members/carers of people who use FES, a further group with FES clinicians and a final group with FES commercial providers and researchers/developers of FES. Due to difficulties in achieving participant numbers on specific data collection days, the procedure was adapted to enable further individual telephone interviews.

FES service users and family members/carers were invited to participate in a focus group by a UK-based National Health Service FES Service. Eight FES service users were invited who had different diagnoses, including Stroke, Spinal Cord Injury (SCI) Multiple Sclerosis (MS), Parkinson’s disease (PD), Traumatic Brain Injury (TBI) and adult Cerebral Palsy. FES service users were invited to bring a family member/carer with them to attend a parallel focus group. Potential participants were identified by a member of the project team based in the FES Clinic (CP). If interested, they were posted an information sheet about the project and provided with the opportunity to ask questions.

Up to eight physiotherapists were invited to participate in a focus group, all linked to the FES Clinic. They were identified and approached by email or in person by a member of the project team based in the FES Clinic (CP). If interested, they were provided with the information sheet and given at least 10 days to decide whether to take part. Potential participants could ask any questions of members of the clinical team. If interested in taking part, they were invited to the focus group meeting.

The aim was to recruit up to eight people involved in development, research and commercial provision of FES through the International Functional Electrical Stimulation Society UK (IFESS). Researchers and commercial providers were identified and approached by email (TS), then provided with an information sheet and a chance to ask questions if interested in participating.

Schedules were used to guide the focus groups and individual interviews (Supplementary 2). Focus groups with FES-users, family members/carers and NHS Physiotherapists took place at the FES Clinic but in order to enhance credibility and trustworthiness, these were led by a researcher who is knowledgeable about FES but independent of the FES Clinic and who did not have a conflict of interest in relation to any specific FES device/company (CB). Written informed consent was taken prior to the start of the focus group and the individual interviews. An observer was present to provide assistance if required by a participant, with a break-out room available for use if a participant required to stop or take a break. Participants were informed that they could take a break from or stop participating at any time without any impact to their care. Focus groups and interviews were audio recorded to record the findings.

All participants were assured of anonymity and data protection. Participants were asked about specific characteristics before the audio-recorder was turned on, to enable generalised descriptions of the group while keeping identities confidential (including: age, gender, medical condition, years since diagnosis, and previous use of FES devices). For family members/carers we also collected age and further details that relate to the person in their lives who uses FES. Physiotherapists were asked about their role, years of experience and previous use of FES devices.

Data collected from the focus groups were transcribed verbatim and analysed using a framework analysis [17] to a) identify the emerging themes with regard to issues relating to FES devices and related service provision for rehabilitation of walking, b) relate these themes to the context of the speaker (patient / family member / therapist characteristics), and c) to identify key questions that need further consensus-development. Two researchers were involved in analysis (CB, CM) to enable discussion of the meaning of themes and add rigour. We analysed the data from a post-postivist perspective, aiming to describe commonalities in what people experienced and thought in relation to FES and its service provision. The framework used related to topics within the interview schedule which broke down into stages of service provision, including: hearing about FES, gaining access to FES, being assessed and monitored over time. The analysis is summarised in supplementary files to increase auditability and therefore rigour – showing the framework of themes used and examples of data within each theme.

## Results

This section summarises participant responses and characteristics, followed by results relating to each of the objectives in turn. Results from quantitative and qualitative data collection are synthesized.

### Participant characteristics

There were 294 entries in the online survey and after data cleaning, responses from 223 people remained (age: mean 43; SD 8.5; 200 Female; 23 Male). Not all included responses to every question. Two hundred five respondents were from the UK and other nationalities included Irish, Polish, Portuguese, New Zealand, Indian, Greek, Dutch, Brazillian, American, and mixed nationality. More people were working in England (186) at the time of survey completion, with 16 in Scotland, 11 in Wales, and four in Northern Ireland. One person was in the Isle of Man, and five people in Ireland. Respondents were predominantly in the advanced/specialist/senior physiotherapist category (*n* = 122) or a band 6/7 physio (*n* = 71). Respondents were predominantly in an NHS FES role (*n* = 137) as opposed to private (*n* = 43) and 160 respondents had completed a specific FES training programme. The ODFS pace was the most commonly used device (*n* = 184) followed by the Bioness L300 (*n* = 52) and Ottobock (*n* = 40), Walkaide (*n* = 29), Actigate (*n* = 10), Stimustep (*n* = 6), XFT2001 (*n* = 1), and other (*n* = 32).

Most respondents viewed the development of FES clinical guidelines for service delivery as very important (151) or somewhat important (67) with the remainder staying neutral or leaving blank (5).

Six people who used FES for different reasons participated in the ‘patient’ focus group. Five used FES to help them walk- two people following a stroke, two due to multiple sclerosis, and one person due to hereditary spastic paraplegia. One person was a wheelchair-user and used FES to help them exercise and walk short distances, following a SCI leading to paralysis from the cervical spine down.

Only one carer was able to attend the focus group held in parallel with the focus group for FES-users; his wife had used FES for about 3-4 years due to MS. In addition, two further individual telephone interviews were held with carers. Both were carers for their husbands, who had both used FES for several years due to MS.

Four people took part in the focus group with physiotherapists. One was a Band 6 Physiotherapist who worked in a rehabilitation day unit, one was a specialist neurological physiotherapist, one was a neuro-physiotherapist working on a stroke ward in a hospital, and the fourth was a pre-registration MSc student who was on a FES-specific clinical placement.

Three further telephone interviews were carried out with a specialist FES lower limb researcher, a specialist FES service provider, a specialist Physiotherapy and FES service provider and one commercial FES provider.

### Objective 1: Exploring the value of and need for clinical guidelines for service provision, including potential use

Survey participants completed a Likert scale in relation to how important they thought clinical guidelines in this area were. Table 1 summarises responses, charted against responses to a question about how people might use such guidelines in their current work and/or role. A substantial 98% of respondents saw clinical guidelines as very or somewhat important. The most strongly endorsed uses of such guidelines include: ensuring best practice and supporting people seeking access to a FES service. Advocating for service funding and informing service development were also seen as very important by many.

**Table 1:** Perceived importance of clinical guidelines focusing on functional electrical stimulation to support walking (Likert Scale) *n*=222 responses

**Within your current work and/or role, how might you use these clinical guidelines? (please tick all that apply)**	**Response frequency in each Likert Scale response category (n)**				
	Very important (*n* = 151) (68%*)	Somewhat important (*n* = 67) (30%*)	Neutral (*n* = 4) (2%*)	Not very important (*n* = 0) (0%*)	Not at all important (*n* = 0) (0%*)
To inform directions for research and development	60	16	0	0	0
To facilitate sales and marketing	5	3	0	0	0
To support a new service design	64	17	0	0	0
To advocate for existing service funding	84	44	0	0	0
To inform development of / benchmark an existing service	89	29	1	0	0
To support people seeking access to a FES service	138	56	1	0	0
To ensure you are following / are aware of best practise	144	59	4	0	0
Other? Please specify #	4	1	0	0	0

^*^ 1 survey respondent did not answer

% frequency given relates to respondents to the question

The qualitative data provided a great deal of support for the value of FES, and its positive impacts on people’s lives, across all stakeholders – summarised in Supplementary 3. All participants reported positive impacts of FES and indicated this was an important topic to explore. As the Expert FES Distributor stated: “I think we’re right to have FES guidelines for foot drop. I think we should start there.”

People explained their reasons for valuing this, by describing life-changing impacts. These included implications of increased ability to mobilise and reduced risk of tripping and falling, as well as reduced fatigue on their confidence, independence, participation in life and quality of life. FES-users described a belief that they would not be able to continue to live their lives and do what they do without FES. Across stakeholders there were also descriptions of the positive impacts extending to affect family and carers. A Physiotherapist Specialist Service Provider commented on how much FES saves the NHS financially through reduction of falls.

Across different stakeholders, reported impacts were described as varying in nature for people with different neurological conditions. This was particularly evident when comparing degenerative versus non-degenerative conditions. Some FES-users with the latter were interested in the small incremental improvements in their performance, whereas those who had degenerative conditions were more focused on prolonging their ability to walk. According to Physiotherapists, FES would be used for different reasons according to a person’s diagnosis, also emphasising the importance of preventing deconditioning through inactivity for people with degenerative conditions. Physiotherapists also reported that for people who have decreased active range of movement, FES enables them to stimulate muscles and achieve passive movement which would otherwise not be possible. An interviewee who is an Expert FES Distributor with experience of different types of FES explained: “I typically hear from MS patients, “Wow, I can walk so much further,” or from a stroke patient, “It reduces my risk of falls.””

It is important to note that some negative impacts of FES were described by FES-users and family members/carers. FES-users critiqued the usability, design, and burden of getting their FES on and off, as well as some effects such as skin irritation and redness from long-term use of the electrodes and deterioration of the electrodes over time. Carers were more likely to discuss negative impacts of the device in relation to its appearance, primarily limiting clothing choices with impacts on confidence and self-esteem. All FES-users felt that benefits of using FES outweighed the disadvantages and reported that they were accepting of the current design, usability and side effects. The Researcher who was interviewed reported that a recent unpublished survey conducted regarding FES use found that patients weigh up the benefits and burden in light of their individual circumstance and decide if they want to use FES based on the benefits and drawbacks that they experience.

The synthesis of findings relating to Objective 1 support the value of FES to people who use it and other people in their lives. Survey responses indicate a clear sense of the importance of developing clinical guidelines in relation to service provision amongst stakeholders.

### Objective 2: Exploring views on the ideal content of clinical guidelines relating to service provision

#### Survey responses

Respondents were asked about how important they thought it was that specific topics and information were included in a clinical guideline. Results are summarised in Table 2. Strongly agree or somewhat agree were selected by between 87 and 99% of respondents for all items, indicating consensus agreement that the items were all important for inclusion. Those achieving highest percentage agreement were: current research evidence and consensus relating to impacts of FES, and evidence relating who is most likely to benefit, and optimal service provision as well as pathways to access this.

**Table 2:** Perceived importance of specific topics and information for inclusion in clinical guidelines focusing on functional electrical stimulation for walking (Likert Scale) *n*=223 responses

6363 How important is it that a clinical guideline includes information about the following	**Response frequency in each Likert Scale response category (n)**				
	Very important	Somewhat important	Neutral	Not very important	Not at all important
Current research evidence and consensus opinion relating to the impacts of FES	163	57	1	1	
Current research evidence and consensus opinion relating to who is most likely to benefit from FES for walking	160	59	3	0	1
Optimal pathways for people to hear about FES as an intervention with potential benefit for their walking	89	106	26	1	1
Optimal pathways for people to gain access to appropriate FES provision	146	68	7	0	1
Appropriate service provision relating to FES to support walking	141	73	7	0	1
Optimal support to start using FES to support walking	129	83	9	0	1
Required / ideal levels of training for providers of FES before starting to use with people to support their walking	112	95	14	0	1
Optimal funding mechanisms for people who use FES to support their walking	106	90	24	0	1
Inclusion of research evidence or consensus about FES as an intervention for other needs than walking	101	95	18	6	1

The possible areas for inclusion were considered in greater depth in the qualitative study. Analysis led to development of themes that are described below, with illustrative quotations included in Supplementary 3. Interpretation of each theme raised questions needing further evidence and/or consensus development, summarised in Supplementary 4.

#### Impacts of FES

This topic was addressed in greater detail in relation to the first objective. When considering the clinical guideline content, The Specialist FES Service Provider who was interviewed felt that although very important, it may be more appropriate to include information about the positive impacts of FES as an introductory section rather than as a specific guideline. The Specialist Service Provider also reported that for clinical guidelines it would be easier for a reader not to differentiate between different conditions within guidelines and to refer to the guidelines as broadly for all upper motor neuron conditions.

#### Holistic use of FES within service provision

A theme that emerged in the qualitative interviews related to “holistic use of FES.” Many FES-users reported that FES had additional benefits to their lives if it was used in combination with exercise and/or exercise classes, describing a desire for greater access to this. Physiotherapists also reported that FES could be used for patients who had limited active movement and could provide a passive way to achieve movement and exercise. They explained that the ability to see passive movement and to exercise using FES could have psychological benefits to patients in addition to the stimulation and movement of their muscles. Two of the three Carers interviewed discussed the possibility of a form of support focused on their ability to support the person using FES. A form of discussion group, online or in person, might enable people with experience of FES to explain some of the issues they have experienced, what expectations are reasonable, and strategies they have used.

The Physiotherapy Specialist Service Provider indicated that since starting their service it has been very important to them to be holistic. This includes ensuring the funding model allows for FES to be applied to different parts of the body as needed, which has led to interesting discoveries. Through trying using FES on the abdominal muscles, the team has found possible application in relation to improving bladder and bowel function. The Specialist Service Provider reported that even people who do not find a functional benefit from FES straight away due to muscle weakness can have muscle strength increases through using FES in combination with exercise. The Expert FES Distributor also felt that a multidisciplinary team approach is crucial, as FES can lead to changes that require different forms of intervention to optimise the outcome.

#### Self-management and psychosocial factors as influences on ability to benefit from FES service provision

The next theme focused on the importance of considering self-management and psychosocial factors. FES-users reported that their ability to self-manage their condition through using FES was an important factor which contributed to its positive impacts on their lives. They explained that psychological constructs such as confidence, persistence, determination not to ‘give up’ and feelings of being self-conscious or social stigma due to their neurological condition could impact on their ability to access and use FES. One of the family carers who was interviewed explained that for her husband, getting used to the experience of FES was quite challenging; she felt that it is something people need a process of adjustment to, and that it is important to be aware of that. Most of the other stakeholders also explained that psychological factors such as motivation and the ability to self-manage are key indicators of which patients may benefit from FES. These factors were seen as potentially more important than which specific neurological condition a person has or their level of function. Both Physiotherapists and the Specialist Service Provider indicated that level of social support and social circumstances are important to assess and consider when FES is being offered. The Specialist Service Provider reported that these factors would be important to include in clinical guidelines for FES.

The Physiotherapy Specialist Service Provider explained that there is a minority of people who may appear suitable for FES but who cannot tolerate the sensation. They explained that some people are not cognitively able to use FES, and others are not at a stage in their recovery journey where they are psychologically ready. They indicated that people of a range of education levels can learn to use FES, while unrealistic or negative expectations can be barriers to adjusting to FES.

#### Awareness of FES and signposting to services

Raising awareness of FES and signposting to appropriate services were reported by FES-users as crucial for gaining knowledge about FES, thereby increasing access to FES services. Most participants described a lack of awareness about FES from patients and clinical teams. This meant that people needed to be more proactive in locating and evaluating information about possible strategies; instead they felt it would be better to be informed and referred by General Practitioners, Consultants, or other health professionals. There was concern raised by some FES-users that if awareness was raised too much then it could lead to services being less able to cope with demand. Others noted increased use of social media to raise awareness.

In contrast, Physiotherapists felt it was important that awareness of FES should be limited to rehabilitation professionals to manage expectations because some people have contraindications or cannot benefit from FES.

Suggestions from stakeholders for raising awareness of FES included increased awareness among primary care providers and healthcare professionals, education of physiotherapy students, and wider dissemination of information online and through professional primary care providers.

#### Initial referral to FES services

FES-users reported a wide range of experiences related to their initial referral for FES, some explaining that getting a referral and actually attending the FES clinic were their biggest hurdles. Physiotherapists reported that they could receive referrals to the FES clinic from any Allied Health Professional or Medical Consultant. Some indicated that they felt self-referral would be an ideal service to offer patients who have been made aware of FES. Others had contrasting views that self-referrals could cause more problems with FES services such as financial and resource barriers. The Specialist Service Provider explained that the rules for referral vary widely between local Clinical Commissioning Groups and they would prefer a scenario where any healthcare professional could refer a person for NHS-funded FES treatment. They reported that the pathway for each condition would vary in terms of referral and indicated that acute and chronic NHS pathways would have different referral systems. This was seen as an issue where guidelines would be valuable.

#### Access to FES services

Issues were raised by FES-users and Physiotherapists that people had minimal access to local FES services across the UK and often had to travel to the specialist service, which could form a barrier. Some participants felt that the ideal would be local provision with support from a Specialist FES Centre. FES-users described feeling part of a ‘post-code lottery’ in terms of who could and could not access FES services. Some people described experiencing ‘battles’ to get through the door to the FES service and that their experience of getting access was not easy, while others felt well supported when trying to access FES. These experiences differed depending on where the person lived. Lack of equipment provision was a key factor in giving access to FES and the Physiotherapy Specialist Service Provider indicated that people’s funded access to FES through the NHS is highly variable, and requires negotiation with Clinical Commissioning Groups. The Expert FES Distributor felt that only around 5% of people who could benefit from FES receive it, due to financial barriers, lack of adoption by service providers due to budget, lack of time and training, and lack of knowledge of the evidence.

The Specialist Service Provider reported that in an ideal service people with all upper motor neuron conditions should be treated the same. Unfortunately funding and decisions about access varied by condition due to the available evidence base, despite clinical guidelines (NICE, 2009) referring to all upper motor neuron conditions.

#### Assessment and treatment by FES services

Both Specialist Service Providers described detailed assessment processes, ensuring people meet selection criteria for FES, without being too prescriptive. One person explained that assessment is crucial to find out whether people can tolerate FES and whether it will help them. They check whether people are cognitively and emotionally able to adjust to using FES. They emphasised that inclusion criteria for using FES are relevant across conditions, giving the examples of cognitive and communication abilities, spasticity, and whether the person has a pacemaker. They indicated that sometimes a person may need strengthening exercise before they can make effective use of FES, due to years of deconditioning. Assessment, fitting and education on using FES was described as rquiring 90 minutes, with a further appointment after six weeks to adjust the settings. Further review takes place after three months, with another review after six months, after which annual review is normal – all with recording of outcome measures. The second Provider included similar elements, but their service runs the first session over two days in order to reduce the level of information a patient must learn over a single day. During this time they teach patients how to use their device and assess their ability to use their device independently.

#### Follow-up of people with FES devices

FES-users, Specialist Service Providers, Physiotherapists and the Expert FES Distributor described an ideal process of three-month and six-month reviews after their first fitting of FES and then an annual ‘MOT’. FES-users valued the time and specialist attention given to these appointments, particularly in relation to their patient-centered, holistic, and personalised nature. FES-users and carers also particularly valued having access to the FES Service by telephone at all times, enabling access to new equipment in the event of a technical problem or replacement being required. The Physiotherapy Specialist Service Provider explained that this annual review is very important as people are ageing, and their needs are changing. She explained that while important, it does not happen everywhere.

#### Measuring progress and outcome measures

All participants discussed the importance of measuring progress at each appointment, including functional ability (e.g. ten-metre walking speed, steps) with and without FES and also their quality of life, pain, employment and independence. The Physiotherapy Specialist Service Provider explained that it is a careful balance between the desire to monitor numerous outcomes versus the importance of focusing on the person’s needs within the time available for their appointment. In their service measures are used to assess tripping, confidence and effort of walking, quality of life, spasticity, pain, and associated reactions. Motion analysis is used by the Researcher as well as clinical outcome measures, and subjective patient-reported outcomes which include the carry-over to daily life. The Expert FES Distributor explained that while improved gait speed is often seen as a valuable outcome this may not reflect improved quality of walking with reduced physiological cost. They would like to see more research into physiological cost and overall physical activity levels with and without FES, as well as actual usage of the device.

#### Clinical training and knowledge of FES service providers

All participants valued clinical training and knowledge and expertise in the people providing FES. Approximately one day of training was suggested as a minimum level initially, but there was discussion about the need for update training and regular use of FES. FES-users indicated that they would be concerned about seeing health care professionals with less understanding and expertise of FES. Physiotherapists and Specialist Service Providers suggested that a one-to-two day training course provided the foundations for using FES, and that ‘hands-on’ experience with devices and different patients were the key factors to increase confidence when using FES with the widest variety of patients. They felt that training could become restrictive if it was too specific and prescribed, making it too difficult for people to be able to use and offer FES to their patients. They reported that peer support, access to specialist knowledge when needed, combined with continuing professional development sessions and practical experience, were the ideal strategies to enable Physiotherapists to use FES.

The Physiotherapy Specialist Service Provider explained that people need a foundation for using FES of around six years as a physiotherapist, including around two years of neurological specialism. They estimated that it takes daily use of FES for around six months to become more confident in using it for people with different needs. A ‘FES User Day’ is arranged annually for people who provide FES to meet and share best practice. The Specialist Service Provider and Expert FES Distributor both mentioned the risks of lack of training leading to FES developing a negative reputation due to poorer outcomes. The Researcher identified a lack of access to training courses in much of the UK.

## Discussion

Key findings from the qualitative and quantitative studies include the value placed on clinical guideline development from numerous and varied stakeholders. When exploring the views of FES-users, their family and carers, there was consistency with previous qualitative research, which supports the transferability of their accounts [4, 18, 19]. The current data supported previous studies that show people to weight the negative and positive impacts of FES when decision-making and elucidated possible barriers to FES use, including lack of funding, distance to reach the specialist service, contraindications, and issues that relate to a person’s ability to self-manage using FES. Interestingly, several of these relate more to service provision than to FES as an intervention. There is a lack of previous research for comparison with the accounts of other stakeholders, although one Canadian study [14] found that over half their online survey respondents would like to use FES more, but experienced similar barriers to access and resources. This resonates with comments from all stakeholders in our qualitative analysis and survey findings that people felt clinical guidelines relating to service provision were important to help increase access to FES. An earlier study [20] also identified resource constraints affecting Irish physiotherapists in relation to FES provision. They advocated greater knowledge exchange between FES stakeholders, which we are hoping to achieve through our ongoing work.

The qualitative data provided some valuable insights into the importance of holistic service provision and of designing this into the wider service design and funding model. This will affect the potential to provide a highly valuable person-centred approach, but is influenced by highly complex relationships between differing local funding mechanisms and the available remit of the FES service. Negotiation of funding would ideally consider the numbers of people likely to benefit, the different clinical conditions represented, the cost of initial equipment as well as maintenance of this along with use of consumables over time. Ideally service funding should support an optimal pathway from initial assessment and treatment through ongoing follow-up. An interesting finding was that although people different clinical conditions may benefit in different ways from FES, this was not seen as a criterion for who could benefit or for referral and service delivery. The potential for benefit was thought to be affected by a variety of other factors that span conditions.

It is complex to develop clinical guidelines, and it may be most practical to develop clinical guidelines focusing on walking, and others focusing on the upper limb, for example. There is a risk, however, that this leads to specification in funding models that a service can only address the lower limb or walking, and therefore cannot provide holistic provision. People with upper motor neuron conditions are highly likely to have concerns relating to, for example, their arm function. They may also have need for increased physical resilience to enable them to benefit from FES. Physiotherapists are autonomous professionals who assess the whole person [21] and service models should allow for them to explore different ways of supporting the person. This is a challenge in relation to current arrangements for service funding and delivery, but one that service providers must engage with.

Most of our interview/focus group participants were associated with specific services, and consequently there were more similar descriptions of optimal assessment, treatment, follow-up and monitoring. This could be seen as a limitation of the study. These are highly established services, however, which can also be seen as having valuable insights into best practice from both published evidence and ongoing practice. This raises the lack of geographical diversity in the participants in both qualitative and quantitative data collection. The online survey included people from different parts of the UK, although there was a clear majority of those living in England. There was also a relatively low response rate from the ACPIN membership, which simply reflect the numbers who felt they could meet the inclusion criterion relating to experience using FES. Qualitative data collection was very focused in the Midlands, with some stakeholders included from further afield.

Current guidelines point to the need to consider FES as an intervention/treatment for people with foot-drop [8, 9] but do not provide the detail that can support a person in optimising the quality and equity of FES delivery, to advocate for FES, or to design a new service. The online survey data gave valuable detail about the value that stakeholders place on clinical guidelines to support them in such developments. Data analysis has raised questions about what might or might not be included in clinical guidelines. Some of these questions have been addressed in previous research, while several have not been investigated, such as optimal follow-up procedures. The lack of specific evidence in relation to some questions means that development of guidelines would require knowledge of expert consensus.

This means that it will be valuable to conduct a consensus development study using an established process, such as a Delphi Consensus study [22] as a further step forward in developing guidelines. This should be informed by a review of the available literature – this is ongoing in the form of an overview of systematic reviews, which is almost complete. The protocol has been published [23]. In moving forward we are using principles within established clinical guideline development processes [24].

## Conclusions

The qualitative and quantitative data in this study have provided clear support from numerous and diverse stakeholders in relation to the value of developing clinical guidelines relating to service provision of FES to support walking. This is seen as important to support optimal service design and to advocate for service provision. Participants in the qualitative data collection prioritised the provision of a service that does not differentiate between people with different clinical conditions, and that allows for flexibility in the use of FES. They also raised the importance of carefully considering and negotiating funding models that allow appropriate service design and potential to provide a holistic person-centred service. This may be facilitated by clinical guideline development. It is hoped that a more detailed clinical guideline would also enable more equitable FES provision across the UK over time by supporting people in advocating for service development. Data analysis and interpretation led to the development of questions that need to be explored through evidence synthesis and consensus development. A Delphi consensus study, informed by evidence synthesis, is being planned based on the findings.

## Supplementary Information

**Additional file 1: Supplementary 1**. Information, Consent and Online Survey Content

**Additional file 2: Supplementary 2**. Schedules for Focus Groups and Individual Interviews

**Additional file 3: Supplementary 3**. Summary of Thematic Analysis: Themes and Illustrative Quotations

**Additional file 4: Supplementary 4**. Summary of Questions Resulting from Analysis to be Used in a Delphi Consensus Study when Developing Clinical Guidelines

## Data Availability

The datasets used and analysed from the anonymous survey are available from the corresponding author upon reasonable request. The datasets gathered from the focus groups and interviews are not available due to the risk of contributors being identified and the potentially sensitive nature of some of the information discussed. A summary of the thematic analysis, themes and illustrative quotations from the focus groups and interviews is provided in Supplementary 3.
