# Supplementary material for: Development of clinical guidelines for service provision of functional electrical stimulation to support walking: mixed method exploration of stakeholder views
Source: BMC Neurol. 2021 Jul 5;21:263. doi: 10.1186/s12883-021-02299-1 (PMC8256555; doi:10.1186/s12883-021-02299-1)
Supplement: Supplementary file 1 — Additional file 1: Supplementary 1. Information, Consent and Online Survey Content [file 12883_2021_2299_MOESM1_ESM.docx]

**Supplementary 1: Information, Consent and Online Survey Content**

**Development of Clinical Guidelines for the use of Functional Electrical Stimulation to Support Walking: Mixed Method Exploration of Stakeholder Views**

*Bulley C, Meagher C, Street T, Adonis A, Peace C, Singleton C, Burridge J.*

**Supplementary 1: Information, consent and online survey content**

**Exploring the Views of People Involved in Developing, Researching and Providing Functional Electrical Stimulation for Walking:**

**Online Survey to Support Development of a Clinical Guideline**

*Page 1 of online survey:*

**What is this survey about?**

This survey is being conducted by a team of researchers working with the UK Association of Chartered Physiotherapists in Neurology. We are in the early stages of developing clinical guidelines for the use of FES to support walking. We are interested in your views because you have important experience of FES and related services.

This survey aims to find out what you think about the potential value of clinical guidelines in this area, how you might use and access clinical guidelines, and what you see as priority areas for inclusion in clinical guidelines. We also give you the opportunity to contact us if you would like to hear about or be involved in further stages of this work.

**How does the survey work?**

This is a research study, so it is important that you know what is involved. After reading the information below, if you wish to learn more, please contact us at the contact email address provided.

The survey is online, and you need to complete this on a laptop or computer. It will take around 10 minutes to complete.

**Who would we like to take part?**

We welcome your views if you are aged 18 or over, feel able to read and respond to this survey in English, and have been involved in development, research, or provision of FES (as a commercial provider or health provider) to people to support their walking.

You do not have to take part. If you wish to, we will ask you to click on the appropriate option to say you consent to completing the survey and that you have received information about the survey in an appropriate way. If you consent and start to complete the survey you must answer all of the questions if you wish to submit, individual questions cannot be skipped. You can stop at any time without giving a reason by clicking the x at the top of the screen to exit the survey. When you click on ‘submit’ at the end of the survey, your responses will become part of our anonymous database.

The survey will take some of your time, but we don’t think you will find any questions upsetting. We have made sure that your responses will be anonymous so that no one can link your responses to you.

**What happens next?**

This survey may not benefit you directly, but we hope that it will help us progress development of a clinical guideline to increase the quality and equity of FES service provision. We will store the survey responses in a password protected server space for ten years after we have finished the survey and will write this up for a research journal and use it to inform further work. You will not be identifiable in any reports.

**Who is involved in this survey?**

This survey is being carried out by a wider research team which includes academic staff from Queen Margaret University, Southampton University, and the ACPIN Working Group. Students at Queen Margaret University are involved in data collection and analysis to support their development and contribute to ongoing work.

The study has been reviewed by an ethics committee in Queen Margaret University Edinburgh and given a favourable ethical opinion.

If you have any further questions about the study, please do not hesitate to contact:

Cathy Bulley at [cbulley@qmu.ac.uk](mailto:cbulley@qmu.ac.uk) (Reader in Physiotherapy at Queen Margaret University)

If you would like to discuss this study with someone independent of the study, please contact:

Judith Lane at jlane@qmu.ac.uk

Thank you for taking the time reading this information.

*Page 2 of online survey:*

***Consent: items to confirm within the online system:***

- I confirm that I have read and understand the participant information for this survey.
- I confirm that I have been provided with the option to contact the research team for more information should I wish to.
- I understand that my participation is both voluntary and anonymous and that I am free to withdraw at any time without giving any reason, without my legal rights being affected.
- I understand that relevant sections of the data collected during the study may be looked at, and audited, by individuals from the Sponsor (Queen Margaret University), where it is relevant to my taking part in this research.
- I understand that once I have submitted my responses to this anonymous online survey, it will not be possible to extract my data.
- I agree to my anonymised data being used in future studies.
- I confirm that I am at least 18 years of age.
- I agree to take part in the study outlined.

Having read the above information:

- I agree with all the above statements

*If clicking agree – the participant will progress to the survey questions.*

- I disagree with one or more of the above statements.

*if clicking disagree – the participant will be thanked for their interest and will not progress to the survey questions Page 3 of online survey:*

***We are interested in finding out about you, to help us when analysing the survey:***

- What is your age (years)?
- What is your gender? Male / Female / Non-binary
- What is your nationality? [open response]
- Where are you currently working (country)? England / Scotland / Ireland / Wales / Other – please state
- What is your current job/role? [open response]
- When thinking specifically about your experience with FES, which of the following describes you best? Options: FES developer / FES researcher / FES provider: NHS based / FES provider: private / FES Commercial provider / Other – please specify
- Do you feel any of the following descriptors also apply to you? (tick all that apply) Options: FES developer / FES researcher / FES provider: NHS based / FES provider: private / FES Commercial provider / Other – please specify
- What qualifications / training have you done to prepare you for working with FES? Options: training programme / undergraduate degree / postgraduate degree / other – please specify
- Which type(s) of FES have you worked with? Tick all that apply: ODFS® PACE / Bioness L300 / Walkaide / XFT2001 / Ottobock / Actigait / Stimustep / Other – please state

**We are interested in your views on developing Clinical Guidelines for FES to support walking:**

- How important do you feel it is to develop clinical guidelines in this area? Likert Scale: Very important – somewhat important – neutral – not very important – not at all important
- Within your current work and/or role, how might you use these clinical guidelines? (please tick all that apply)
  - To inform directions for research and development
  - To facilitate sales and marketing
  - To support a new service design
  - To advocate for existing service funding
  - To inform development of / benchmark an existing service
  - To support people seeking access to a FES service
  - To ensure you are following / are aware of best practise
  - Other? Please specify
- What is the best way for you to hear about a new clinical guideline? Please click all that apply:
  - Professional Group (which)
  - Networking group (which)
  - Social media (which type?)
  - Conferences (which?)
  - Staying involved or being updated directly (see below)
  - Other (please specify)

We are interested in your thoughts on what topics and information are more or less important to include in a clinical guideline. Below are some possibilities: for each one we ask you how important you think it is. We also give you the opportunity to suggest other possibilities.

- How important is it that a clinical guideline includes information about the following?
  - Current research evidence and consensus opinion relating to the impacts of FES
  - Current research evidence and consensus opinion relating to who is most likely to benefit from FES for walking
  - Optimal pathways for people to hear about FES as an intervention with potential benefit for their walking
  - Optimal pathways for people to gain access to appropriate FES provision
  - Appropriate service provision relating to FES to support walking
  - Optimal support to start using FES to support walking
  - Optimal long-term support for FES to support walking
  - Required / ideal levels of training for providers of FES before starting to use with people to support their walking
  - Optimal funding mechanisms for people who use FES to support their walking
  - Inclusion of research evidence or consensus about FES as an intervention for other needs than walking
  - Other: Please specify…

Likert scale for each item:

Very important 🡪 Important 🡪 Neutral 🡪 Not very important 🡪 Not at all important

- For any of the items above, please use the space below if you wish to expand on your answer.

**Thank you for your time in completing this survey – your views will be used to inform the next steps. If you wish to be kept informed of further developments and/or to be involved in the next steps, please email Cathy Bulley directly on** [**cbulley@qmu.ac.uk**](mailto:cbulley@qmu.ac.uk)**.**
