# Supplementary material for: Development of clinical guidelines for service provision of functional electrical stimulation to support walking: mixed method exploration of stakeholder views
Source: BMC Neurol. 2021 Jul 5;21:263. doi: 10.1186/s12883-021-02299-1 (PMC8256555; doi:10.1186/s12883-021-02299-1)
Supplement: Supplementary file 2 — Additional file 2: Supplementary 2. Schedules for Focus Groups and Individual Interviews [file 12883_2021_2299_MOESM2_ESM.doc]

**Supplementary 2: Schedules for Focus Groups and Individual Interviews**

**Development of Clinical Guidelines for the use of Functional Electrical Stimulation to Support Walking: Mixed Method Exploration of Stakeholder Views**

*Bulley C, Meagher C, Street T, Adonis A, Peace C, Singleton C, Burridge J.*

**Supplementary 2: Schedules for focus groups and individual interviews**

**Introduction to each focus group:**

I represent a team of people who are working on developing a clinical guideline for best practice when using FES to support walking in people with leg weakness. Clinical guidelines can help influence decisions on funding services or treatments and how they are delivered. It is very important that people who use clinical guidelines are able to influence their development.

We have asked you to come to this focus group because you have important experience of FES [tailor to the group] and can give us your thoughts on what a clinical guideline should include and how and with whom it should be used to ensure all patients receive a high standard of effective care.

Starting off

- You are the experts and we are interested in your experiences.
- We have some topics we will bring up but there may be others we have not thought of and it will be great to hear about these
- Please protect confidentiality for each other in the group
- Please respect each other’s views, these may be different as experiences vary
- We need to type up everything that is said so that we can analyse it carefully, this will be easier if people are able to speak one at a time.

**1) Topic guide - PATIENTS**

Firstly, could we introduce ourselves to one another and give some background to the experience you have with using FES?

A clinical guideline gives important information to you and to your therapist on the best way to use FES and support someone using FES.

- Firstly, we are interested in how FES affects people and their lives:
  - Could you tell me more about what you think people and their therapists should know?
    - Possible prompts: How has life changed since starting to use FES? Do you feel it has any impacts on your mobility / walking / getting out of the house / being involved in activities that you are interested in?
    - Do you feel it affects the way you feel / or the way you feel about yourself?
    - If you were talking to someone who was thinking about using FES, what would you say are the challenges / things that make it more difficult?
    - Do you have any suggestions about things that make it easier?
  - Having heard other people’s experiences of using FES, do you feel that you and others have similar or different experiences? Do you feel this is affected by the reason you have foot-drop?
- We are interested in how people accessed FES.
  - how did you first hear about / find out about FES?
    - What did you think of that?
    - What would you have preferred / what do you think would be best?
  - When you tried to find out if you might benefit from FES, how did you find this process?
    - What happened next – how did you get referred?
    - What did you think of that?
    - What would you have preferred / what do you think would be best?
  - Having heard other people’s experiences of using FES, do you feel that you and others have similar or different experiences? how people should be assessed and treated with FES?
- We would like to hear more about your appointments and follow-up.
  - When you had your first appointment, what was it like?
    - What did you think of that?
    - Would you have wanted anything done differently?
  - Have you received follow-up appointments after getting FES for the first time?
    - How often?
    - What were they like?
    - What did you think of that?
    - What do you think would be best?
  - when you have come back for follow-up appointments, has the physiotherapist taken any measurements or asked questions to find out if FES is helping?
    - Can you remember what they did?
    - How did you find that?
    - Do you feel these are the things that matter most to you?
    - What do you think people should measure?
  - Having heard other people’s experiences of this, do you feel that you and others have similar or different experiences?
- We are interested in knowing more about how much training you think physiotherapists should have before they provide FES.
  - Have you had any experiences that affect what you think about this?
  - What do you think would be best?
- If you are willing to discuss this, who paid for your FES device? How was if funded?
  - What do you think of that?
  - What do you think would be best?
  - Having heard other people’s experiences of this, do you feel that you and others have similar or different experiences?

**2) Topic guide - FAMILY and CARERS**

Firstly, could we introduce ourselves to one another and give some background to the experience you have with supporting someone who uses FES?

A clinical guideline gives important information to you and to your therapist on the best way to use FES and support someone using FES.

- Firstly, we are interested in how FES affects people and their lives:
  - Could you tell me more about what you think people and their therapists should know?
    - How has life changed for you and for the person you support since starting to use FES?
    - Do you feel it has any impacts on their mobility / walking / getting out of the house / being involved in activities that they are interested in / that you are interested in?
    - Do you feel it affects the way you feel / or the way you feel about the person you support?
    - If you were talking to someone who was thinking about using FES, what would you say are the challenges / things that make it more difficult?
    - Do you have any suggestions about things that make it easier?
  - Having heard other people’s experiences of using FES, do you feel that you and others have similar or different experiences? Do you feel this is affected by the reason a person has foot-drop?
- We are interested in how people accessed FES.
  - how did you and/or the person you support first hear about / find out about FES?
    - What did you think of that?
    - What would you have preferred / what do you think would be best?
  - When you and the person you support tried to find out if they might benefit from FES, how did you find this process?
    - What happened next – how were they referred?
    - What did you think of that?
    - What would you have preferred / what do you think would be best?
  - Having heard other people’s experiences of using FES, do you feel that you and others have similar or different experiences? Do you feel this is affected by the reason a person has foot-drop?
- We would like to hear more about your appointments and follow-up.
  - When you had your first appointment, what was it like?
    - What did you think of that?
    - Would you have wanted anything done differently?
  - Have you received follow-up appointments after getting FES for the first time?
    - How often?
    - What were they like?
    - What did you think of that?
    - What do you think would be best?
  - when you have come back for follow-up appointments, has the physiotherapist taken any measurements or asked questions to find out if FES is helping?
    - Can you remember what they did?
    - How did you find that?
    - Do you feel these are the things that matter most to you?
    - What do you think people should measure?
  - Having heard other people’s experiences of this, do you feel that you and others have similar or different experiences?
- We are interested in what you think family and carers need to know about FES and whether there should be information in the guideline about this.
  - What do you feel might be the most important information for family and carers to know?
  - How do you think family and carers find this information at the moment?
- We are interested in knowing more about how much training you think physiotherapists should have before they provide FES.
  - Have you had any experiences that affect what you think about this?
  - What do you think would be best?
- If you are willing to discuss this, who paid for your FES device? How was if funded?
  - What do you think of that?
  - What do you think would be best?
  - Having heard other people’s experiences of this, do you feel that you and others have similar or different experiences?

**3) Topic guide - PHYSIOTHERAPY PROVIDERS OF FES**

Firstly, could we introduce ourselves to one another and give some background to the experience you have with using FES?

Do you think a clinical guideline should include information about:

- how FES affects people and their lives?
  - Could you tell me more?
  - Do you feel there are any differences depending on the reason FES is being used? E.g. depending on whether a person has foot-drop due to MS or due to stroke?
- how people should hear about / find out about FES?
  - What do you think would be best?
  - Do you feel there are any differences depending on the reason FES is being used?
- how to work out who might benefit from FES?
  - What do you think would be best?
  - Do you feel there are any differences depending on the reason FES is being used?
  - Do you have views on which patients are most likely to benefit and those that are not?
  - Do you think psycho-social factors are important as well as physical ones?
- how people access services, e.g. how they are referred?
  - What happens in your NHS Trust currently?
  - What do you think of that?
  - What do you think would be best?
  - Do you feel there are any differences depending on the reason FES is being used?
- how people should be assessed and treated with FES?
  - What do you think would be best?
  - Do you feel there are any differences depending on the reason FES is being used?
- what follow-up people should get after they receive FES?
  - What do you think would be best?
  - Do you feel there are any differences depending on the reason FES is being used?
- what outcomes are important to monitor when people are using FES, and how often?
  - What do you think would be best?
  - Do you feel there are any differences depending on the reason FES is being used?
- how people should be trained before they provide FES?
  - What do you think would be best?
  - Do you feel there are any differences depending on the reason FES is being used?
- How FES and FES services should be funded?
  - What do you think would be best?
  - Do you feel there are any differences depending on the reason FES is being used?
