# Supplementary material for: Development of clinical guidelines for service provision of functional electrical stimulation to support walking: mixed method exploration of stakeholder views
Source: BMC Neurol. 2021 Jul 5;21:263. doi: 10.1186/s12883-021-02299-1 (PMC8256555; doi:10.1186/s12883-021-02299-1)
Supplement: Supplementary file 3 — Additional file 3: Supplementary 3. Summary of Thematic Analysis: Themes and Illustrative Quotations [file 12883_2021_2299_MOESM3_ESM.docx]

**Supplementary 3: Summary of Thematic Analysis: Themes and Illustrative Quotations**

**Development of Clinical Guidelines for the use of Functional Electrical Stimulation to Support Walking: Mixed Method Exploration of Stakeholder Views**

*Bulley C, Meagher C, Street T, Adonis A, Peace C, Singleton C, Burridge J.*

**Supplementary 3: Summary of framework analysis: themes and illustrative quotations**

| **Theme** | **Illustrative quotes and participants** |
| --- | --- |
| **Positive impacts of FES on people’s lives** | “I would trip (without it). It Allows me first to get out of the house and walk. At first, I used to trip as my legs gave way. I would fall and couldn’t get up. Now I can get up myself without calling the lifeline.” (Neurological Patient)  “If I didn’t have the FES I wouldn’t be able to do the exercise that I do so the carry-over is that I am more capable to do a full day of work so I find my time to do my exercise but this gives me more energy to do a full working day and to do stuff with the family as well in the evenings and weekends.” (Neurological Patient)  “Yeah I think the device itself is there for a purpose, it has a function and it’s important that she has it. Without it she’s vulnerable. If she removes it there’s a distinct and noticeable difference on how she moves and her mobility.” (Family member 1)  “So, it did take that pressure off, it made it a little bit more enjoyable if we were out and about so that I wasn’t worried about him all of the time and I wasn’t looking ahead for any obstacles in the way.” (Family member 2)  “MS as an example, this is a deteriorating and on-going condition so they are likely to rely on FES more heavily in the future whereas in stroke patients the impact is immediate and you hope that part of their recovery rather than a long term thing.” (Physiotherapist)  “…especially if people are tripping or falling before FES and then afterwards they feel safe and confident I think that’s a huge psychological as well as physical impact. (Physiotherapist).  “there’s not a day that goes by without a patient saying how much it changes their lives. It’s about providing independence, safety of mobilisation, hope that they can mobilise, independence and also that is really important for the carers and family members around them as gives them reassurance and confidence that the individual is able to look after themselves independently.” “Another thing our patients will say is that it allows them to engage and participate rather than be left at home… It’s peace of mind of the carer as well as letting the patient do what they want or need to do without the feeling they are totally reliant on the carer. It’s the whole family including children… In the working environment, there are colleagues around work who would feel anxious if the patient was trying to walk. This means the patient can be more independent at work as well.” “…there are huge cost benefits to the NHS in preventing falls in people.” (Physiotherapist Specialist Service Provider)  “Yeah, well it’s a huge impact for some people… it allows people to go out and go shopping or even… there’s a whole range…. somebody (in our research study) was very fit and actually played golf, that (FES) allowed her to play golf for18 holes.” (Researcher)  “it’s an introduction rather than part of the guidelines as such, because it’s not telling you what you should or should not do.” “….in terms of its orthotic effect, it is very similar across all groups, so there’s good reason I think, to try and keep it as coherent at the motor neuron indication rather than put any doubt into people reading the guidelines’ minds that it’s, you know, it’s as used to stroke or MS”. (Specialist Service Provider)  “…if all of a sudden they can walk in the mall with their loved one or go to their granddaughter’s soccer game or, you know, just walk a further distance with more confidence that they’re not going to fall or look awkward, I think that has a huge psychosocial impact on patients using FES.” (Expert FES Distributor) |
| **Negative impacts of FES on people’s lives** | “I find one of the problems if you’re wearing the electrodes all day as I’m working, I do get a lot of irritation and redness on the skin which affects the functioning of the mechanism and that’s one of the major problems I find with it, as well as siting the electrodes, batteries wearing out and there are a combination of things that can go wrong which is frustrating. …Although you learn to live with it.” (Neurological Patient).  “I couldn’t do my job without it, I would be absolutely no good but as I say for me it’s been a lifeline - it does have its ups and downs but I use different hypo-allergenic electrodes as the other ones caused irritation… It could be a lot worse, so you just learn to deal with it”. (Neurological Patient)  “one of the issues is with clothing and this can be restrictive, she’s found it restrictive in allowing her to wear certain types of clothes she can wear. … it’s also affected her confidence, her self-esteem and you know...” (Family Member 1)  “We did a survey… that’s sort of what came out, some balance of people what they, the benefit and the drawbacks.” (Researcher) |
| **Holistic use of FES** | “Once you’ve got the FES it’s marvelous but feel that you need more a cue to keep your body fit like going on a bike or walking or balancing because I’m doing a lot of balancing exercises which is helpful.” (Neurological Patient)  “I think any advice like that would be welcomed because otherwise it’s going on line or finding a forum that you can get advice from. Other people of a similar age or with similar issues.” “Some people might be struggling to come to terms with it initially would benefit from something like a group session? …I think even having a point of contact here or online that people can be made aware of, and there is a forum where people can blog their own situation and circumstances online for people to read and get a better understanding of.” (Family member 1)  “…I made a conscious decision not to call it a ‘drop foot service’ I would call it the FES service and I kept the specification very broad whenever we were negotiating with the commissioners as I am now able to provide FES anywhere in the body and with any machine. That has allowed me… to treat the patient holistically as well. A stroke doesn’t just affect the leg, it affects the arm as well so we have been treating both. A stroke or an MS patient needs a good solid core so we can treat the trunk and the abdomen and it’s through work like that that you discover other uses and ways that the FES is useful... I’ve used electrical stimulation on the abdomen and the core for core stability and patients were coming back and telling me they were no longer constipated... It should be related to the care that is required for that person.” (Physiotherapy Specialist Service Provider)  “So, I believe that the best clinical outcome is going to involve… FES with physio, with their orthotist, with their neurologist, because as we reduce perhaps physiological cost and we have better spasticity management that could have an impact on their pharmacological regiment. The physical therapist now will be able to attend to other compensations to their gait and perhaps be able to better work on the knee and the hip and the overall posture and gait symmetry in combination with FES.” (Expert FES Distributor) |
| **Self-management and psychosocial factors** | “Some people don’t have the confidence to ask. But I am persistent, sometimes it takes me 5 minutes sometimes half an hour but if I need help, I will say something. I keep on until I do it and don’t give up. I just keep calm about it.” (Neurological patient).  “I think getting used to it. …I don’t think it’s something that you can use for a week and think, yes, this is fantastic, I’m going to keep on with this. It is a process that over a month, I would say, I don’t think it’s something that you can say after a week. And maybe some people think after a week, after two weeks, no, this isn’t for me. But I think you do have to persevere. It’s like anything new, isn’t it, you have to persevere with it.” (Family member 2)  “Someone who is willing to put practice in independently. I was going to say someone who has support.” (Physiotherapist)  “…patients can be so traumatised by the fact that they’ve had a stroke that they are not ready to engage in active rehab or to help themselves… they can come to you saying “you’re going to make me run” so the expectation can be a psychological barrier as well - that you never satisfy them or they haven’t come to terms with the condition they have… where I have some people with high expectations, I also have others who are quite reticent and think it won’t work.” (Physiotherapy Specialist Service Provider)  “…it’s really difficult to define exactly any limit to, you know, which patients would benefit more than others. I mean, there’s the characteristics perhaps like, motivation and willingness to adhere to treatment makes a big difference….a certain degree of cognitive ability.” (Specialist Service Provider)  “if someone…finds it difficult to fit the equipment, often a carer or partner will help them with it. So their social circumstance certainly makes a difference….” (Researcher) |
| **Awareness of FES and signposting** | “It’s interesting to learn how we all got into it via different routes and that reflects on the fact that it’s not well enough known from patients and clinical side… unless there’s the spreading of knowledge and communicating, you’re fighting an uphill battle…” (Neurological Patient)  “I guess there’s a risk that the floodgates open and if the back-up service isn’t able to accommodate that then there’s a danger it might make the situation worse for some people as the waiting list could get longer.” (Neurological Patient)  “She’s very switched on to using the internet and doing research, she doesn’t wait around to be told or asked, she’s very proactive in trying to find easier solutions, or new innovative ways that may be introduced… To begin with, it was a bit confusing as there can be so much information that’s not specific to your requirements so you have to filter it. It’s a case of reading a lot before you realise what you need, like a process of elimination.” (Family member 1)  “…it has to be something that is widely known by everyone that could be dealing with not only an MS patient, it could be suitable for other people. So, doctors, GPs surgeries, consultants, they all need to be on the same page.” (Family member 2)  “I’ve had quite a few patients who have asked about it or their family members have asked about it and it’s been completely inappropriate but hard to explain why. …So from our perspective would be a lot easier if it was us providing the information but that’s not always the case.” (Physiotherapist).  “The knowledge base is very patchy by the professionals… it’s sometimes word of mouth from one patient to another or through the healthcare professionals that they have that are very aware of the FES world...” “From a user’s point of view – if they are in a rehab environment should be aware of all the tools that are available for them to provide rehab for them and FES is one of those tools – and not simply saying automatically, that they will put a splint on.” (Physiotherapy Specialist Service Provider)  “All publicity is welcome to bring in more referrals to our service. So in terms of guidelines, I think you can make a recommendation that it’s a standard piece of information that healthcare providers tell the patients.” (Specialist Service Provider)  “I sometimes think I should really start here, but, with the physiotherapy students… But I think it’s education of physios and consultants at least they’re aware of it, and then the funding and the politics come later maybe.” (Researcher)  “We’re finding different ways to promote our technologies and make appropriate patients aware of our technologies. We’ve done so through physio groups. We’ve done so through neurology and physiatry groups, and we’ve done so through orthotic and prosthetic facilities as well, and now, with the advent of social media, we’re doing more direct-to-patient type educational information, you know, from Facebook and Instagram or what have you... So, I think it has to be a multi-pronged approach to make patients aware of FES technology…” (Expert FES Distributor) |
| **Initial referral for FES** | “Originally, I knew someone… she introduced me to FES and what it could do. I then decided to ask the specialist rehab consultant… and I was referred here... But the point I’m making it’s about the specialists and consultants knowing because if I hadn’t met this woman, I wouldn’t have known to pursue it and I’m quote vocal so I’m prepared to ask but others may not be so much.” (Neurological Patient)  **“**We take referrals from any consultant AHP or Nurse. We don’t take self-referrals at the minute but I think we should in a sense. Anyway.” (Physiotherapist)  “The referral procedure tends to vary on the rules of whichever institution you’re in, or even funding area they’re from. ….it just depends on the local rules. I mean, we also take self-referrals, but we can’t take self-referrals for NHS-funded treatment. It can only be for privately-funded treatment….I guess we would like any healthcare professional to be able to refer in for NHS-funded treatment; that would be the ideal for our service.” “I can’t think there should be any significant difference (between conditions).” (Specialist Service Provider) |
| **Access to FES** | “it’s a shame that it’s so far – it’s about 80 miles for me to get here so I have to organise that… If there was a local specialist in my area, that would be fantastic where I could do a 3 or 6 month or yearly review where I can locally touch base, would be good if it could open up more country-wide that would be amazing.” (Neurological Patient)  “It is a bit of a lottery with the postcodes here.” (Neurological Patient)  “I personally think it’s something that any patient should be able to have without having to pay a fee. To a lot of people it’s a necessity to help improve their quality of life, so to me it’s a bit of a no brainer.” (Family member 1 )  “they might not sign up to the journey to trial something they don’t know.” (Physiotherapist)  “…so ideally, we would like all patients to be treated the same, because there is no, in our experience, they respond in very similar ways so we don’t think there should be differences for different conditions.” (Specialist Service Provider)  “…maybe 5% of the patients that have foot drop currently have an FES device, so yes… I think, one, because of a poor reimbursement environment and the cost… then, two, lack of clinic adoption. So, not all neuro rehab or physio facilities use FES as much as they probably should. Yes, complex. So, I mean budget restraints, training, lack of time, because it does take some effort and time to set up FES technologies, lack of understanding of long-term clinical outcomes.” (Expert FES Distributor) |
| **Assessment and treatment with FES** | “Basically it will either work for them or not so it has to be tried first…. Cognition is a criteria for us...” Researcher: ““Would you say you have different inclusion criteria for people with different conditions?” Physiotherapy Specialist Service Provider: “No.” Researcher: “So they are generic across the conditions?” Physiotherapy Specialist Service Provider: “Yes they are generic. So for example, do they have cognition, can we communicate with them, do they have spasticity, do they have a pace-maker, etc. For me it’s holistic as far as neurological conditions are concerned.” “Yes the main one [contraindication] is that they get an adverse reaction to it.” “I think you should try it. Essentially FES is a very crude nerve conduction test and you cannot find out whether you’re going to get any response to it until you’ve tried it.” “Yes and their response to it because you could have for instance, someone who has completely de-conditioned, for instance a stroke patient who is 4 or 5 years down the line - so when you’re doing your assessment you’re saying to yourself, does this work but also is this patient ready to go straight to walking or do we need to recondition the muscles beforehand and do a programme of exercise first.” (Physiotherapy Specialist Service Provider)  “if you try and be too prescriptive, then you can restrict patients coming in who could benefit for reasons that haven’t been properly considered, so it’s a tricky one. Do a basic physical assessment of the sort of MRC scores…. try the FES, explain all about it, and most of the assessments, we can see immediately if there is an orthotic benefit from treatment and therefore if…and we’ll do basic outcome measures like walking speed, bulk, to see if their foot…to put a bit of a number on whether there’s an immediate effect… we have this assessment and then we ask them back to start treatment. we always, or nearly always set up FES over two days. First day, teach the patient how to use it, second day, assess their ability to do so and give any more help that they need, any more teaching etc.” (Specialist Service Provider) |
| **Follow-up of people with FES devices** | “I had the initial fitting and then I had a 3 month review and then again after 6 months… they compare my movement with and without the FES…they can see the change over the years – they don’t rush, they takes an hour over it and check everyone. Amazing.” (Neurological Patient)  “The yearly MOT as it were, everything is checked... But it’s holistic. It’s not just about the machine, it’s about you as a person and how you’re doing as well and are you still managing or not. It’s not a generic service - we are not an assembly line it’s very much taken off and looked at and from that it’s you and your lifestyle that are considered, about your family life, whether you’re working or not working etc.” (Neurological Patient)  “it’s a very encompassing session when you come here for the check-up. It feels like a personal service rather than just a cookie cutter.” (Neurological Patient)  “If she’s finding difficulty with the device, she’ll call and ask someone.” (Family member 1)  “So, there is somebody on the end of a phone. So, if you’re not sure of something or something is not going well, you can speak to somebody about it.” (Family member 2)  “I have ensured that I have understood what it takes to provide the long-term service and what I need to deliver the type of service that I need. i.e. how many electrodes do I need per patient, what if the machine gets damaged etc. so it’s not only the package of what you give for a fitting but what do they need going forward as consumable and maintenance need. The standard pathway is assessment (what is suitable) and then a fitting and instructions - this takes an hour and a half, as well as outcome measures and then 6 weeks later, because they will have changed by then… we need to look at if they need as much power as they did initially. Three months after that they are reviewed, and outcome measures recorded and six months after that they are reviewed again with outcome measures recorded and then they go to annual review.” (Physiotherapy Specialist Service Provider)  “Our standard follow-up is, so, we have our two set-ups, then we re-assess six weeks later, and then three months after that, and then six months after that and then annually for as long as they use FES….we believe that people shouldn’t be discharged while still using a medical device, you know, people change, devices change, they wear out, so there’s a need to do regular planned follow-up, to ensure safe and effective use.” (Specialist Service Provider)  “So, maybe see the patient after delivery of the device. Maybe two weeks after the delivery, then maybe 60 days after that, and then maybe, you know, three to six months after that, and then maybe once a year. I mean, it could be that infrequently, depending on the patient, yes.” (Expert FES Distributor) |
| **Measuring progress and outcome measures** | “They have my record for the last 10 years and they compare my movement with and without the FES. They can see the change over the years – she doesn’t rush, she takes an hour over it and checked everyone. Amazing.” (Neurological Patient)  “it’s about your independence, quality of life, if it affect pain, mobility – so it’s a very encompassing session when you come here for the check-up.” (Neurological Patient)  “You can’t have 10 outcome measures to do with the patient as it takes up the whole appointment. There has to be a balance – is it a research project or is it a service evaluation to check that you are still doing what you need to do and making a difference to the patient?” “We have a visual analogue scale, which measures tripping, confidence and effort of walking, quality of life, spasticity, pain, associated reaction. There’s a scale for each of these as they are now and we compare against what they were when they started the treatment. And then we have 10m walking distance assessment both with and without the stimulator.” (Physiotherapy Specialist Service Provider)  “We use motion analysis, but sort of my recent theme of thinking is that, well it has to be functional so maybe asking people more about what they can do now, because it’s a bit more softer…. So we can do a ten-metre walk in the lab, and actually they don’t show that much difference” (Researcher)  “Well, we routinely do walking speed as the assessments. And we have tried other assessments, but clinic time is short” (Specialist Service Provider)  “It’s interesting because a lot of times, we look at gait speed… It doesn’t mean that, kinematically, it’s correct. It doesn’t mean there’s a reduction in physiological cost… They’ve just compensated. Now, if we look at other parameters, such as the six-minute walk and the other classical assessments like the Berg analysis and things such as that in combination to look at other functional capacities, and I would like to see in the future more emphasis on reduction in physiological cost… I think compliance and usage is a huge indicator for overall recovery and good clinical outcome.” (Expert FES Distributor) |
| **Clinical training and knowledge** | “in actual fact you do need someone who is a specialist in the field to be able to understand the neurological setting that each person is in. You need a line of specialism...” (Neurological Patient)  “I think the hands on practice is what made me a better practitioner with it. I think you can have the theory, you can be taught it but if you’re not given the opportunity to try and fail, get it in the right place, this is what builds the confidence.” (Physiotherapist)  “I think if you make it too difficult or prescriptive for people to use it then they won’t use it. So why not let the 2 days be enough and then let people have the opportunity to use the machine and get comfortable and confident with it.” (Physiotherapist)  “There’s a knowledge base of neurology which is important so a minimum of about 6 years with at least 2 years neuro experience so they are not overwhelmed by the neurology aspect of the patient in front of them as well as the technology that I’m going to apply to them. As far as the technology is concerned, the training programme is 1 day and I am a trainer as well. If an individual on their own with no support back at base, they have a difficult journey. If it’s a couple of people who are back at the same base they can talk and learn from each other. My team… will tell you it takes them on a daily basis of giving a clinic, at least 6 months for them to get really comfortable with the portfolio of experience of the patients - they are getting to understand the nuances and the differences of each of the conditions and how they use the stimulator. …I feel the follow up is needed 3 months down the line to find out how the training has gone … and for those who don’t have the support back at base, we need to give them a bit more support. …We do try to provide a user day to come together once a year and share best practice. The technology is constantly changing which can also be a problem… The minimum is definitely one day.” Researcher: “If you had a choice, would you have a minimum standard for training or updates?” Physiotherapy Specialist Service Provider: “Yes there should be.” (Physiotherapy Specialist Service Provider)  “...it’s still our policy that, you know, training is required… In terms of CPD, well, the biggest thing with, you know, keeping…being good at FES, is seeing lots of patients. … I think (a guideline) should make recommendations that FES clinicians receive some training…. Saying the amount of training I think is very hard.” (Specialist Service Provider)  “…if you do have a physio that does not use FES very often and they try to programme it and they end up with a poor clinical outcome, well the patient’s not going to buy a device. So, we need to have these clinicians that are not only trained, but use a lot … the more you use it, the better your clinical outcome’s going to be… they would need a six to eight-hour didactic and practical training to get the basic certification. Then I would encourage, as part of the follow-up, that these physios or orthotists would use the technology in a treatment setting, a minimum of six to eight hours a month. …So, they need to at least have one patient in their caseload every month that is using FES, and then three months later, I would like to see an advanced, like, four-hour training to offer more advanced training,.” (Expert FES Distributor) |
