# Supplementary material for: Development of clinical guidelines for service provision of functional electrical stimulation to support walking: mixed method exploration of stakeholder views
Source: BMC Neurol. 2021 Jul 5;21:263. doi: 10.1186/s12883-021-02299-1 (PMC8256555; doi:10.1186/s12883-021-02299-1)
Supplement: Supplementary file 4 — Additional file 4: Supplementary 4. Summary of Questions Resulting from Analysis to be Used in a Delphi Consensus Study when Developing Clinical Guidelines [file 12883_2021_2299_MOESM4_ESM.docx]

**Supplementary 4: Summary of Questions Resulting from Analysis to be Used in a Delphi Consensus Study when Developing Clinical Guidelines**

**Development of Clinical Guidelines for the use of Functional Electrical Stimulation to Support Walking: Mixed Method Exploration of Stakeholder Views**

*Bulley C, Meagher C, Street T, Adonis A, Peace C, Singleton C, Burridge J.*

**Supplementary 4: Summary of questions resulting from analysis to be used in a Delphi Consensus study when developing clinical guidelines**

| Theme area | Questions |
| --- | --- |
| Positive Impact of FES on people’s lives | 1. Should the positive impact on people’s lives be addressed in the clinical guideline document: 2. No, this information is not necessary in a clinical guideline document 3. Yes, it would be valuable to include a brief introduction to the guidelines that comments on the positive impacts of FES on people’s lives 4. Yes, if would be valuable to include a longer section of general information on the positive impacts of FES on people’s lives 5. Yes, there should be a clinical guideline that uses information on the positive impacts of FES on people’s lives, e.g. to recommend all neurological patients should be considered for FES 6. Should clinical guidelines differentiate between use of FES for people with different clinical conditions: 7. No, it is important to focus on the way FES can provide benefit to people who meet more generic criteria that relate to upper motor neuron conditions, and not differentiate between clinical conditions 8. No, but it would be valuable to provide information about how FES can be used in different ways to support people with different clinical conditions 9. Yes, it is important to write clinical guidelines that differentiate between uses of FES for people with different clinical conditions. 10. Specific clinical guidelines should be developed for each clinical condition where FES is applied |
| Negative Impacts of FES on people’s lives | 1. Should clinical guidelines address the potential negative impacts of FES on a person’s life 2. No, this information is not necessary in a clinical guideline document 3. Yes, it would be valuable to include a brief introduction to the guideline document that comments the possible negative impacts of FES on people’s lives 4. Yes, it would be valuable to include in a longer section of general information the possible negative impacts of FES on people’s lives 5. Yes, there should be a clinical guideline that uses information on the possible negative impacts of FES on people’s lives, with information on how to prevent/ assess/ respond to any negative impacts people’s lives 6. Yes, there should be a clinical guideline that uses information on the possible negative impacts of FES on people’s lives, with information on how to reduce and respond to inappropriate referrals. |
| Holistic use of FES | 1. Should clinical guidelines address the provision of FES within holistic service delivery which focuses on the whole person? 2. No, the clinical guidelines should focus on FES as an intervention and not in the context of wider, holistic service delivery 3. Yes, it would be valuable to include in a brief introduction to the guideline document more information on wider, holistic service delivery 4. Yes, it would be valuable to include in a longer section of general information more information on wider, holistic service delivery 5. Yes, there should be a clinical guideline that FES should not be limited 6. Should clinical guidelines advocate for service design which does not limit use of FES to support for walking or to provision for a specific clinical condition? 7. No, the clinical guidelines should focus on FES as an intervention and not address service design 8. Yes, it would be valuable to include in a brief introduction to the guideline document more information on service design 9. Yes, it would be valuable to include in a longer section of general information more information on service design 10. Yes, there should be a clinical guideline that FES service delivery should not be limited to use of FES to support walking, or to provision for a specific clinical condition   6) Should clinical guidelines address the use of FES in combination with exercise / rehabilitation?   1. No, the clinical guidelines should focus on FES as an intervention and not address its use in combination with other interventions 2. Yes, it would be valuable to include in a longer section of general information more information about supplementary interventions 3. Yes, there should be a clinical guideline that provides recommendations on what exercise / rehabilitation may benefit different patient groups 4. Yes, there should be a clinical guideline to recommend that patients should be referred to an exercise class or supported to access equipment   7) Should clinical guidelines address support for family and carers?   1. No, the clinical guidelines should not address support for family and carers 2. Yes, it would be valuable to include in a longer section of general information suggestions about provision of support for family and carers 3. Yes, there should be a clinical guideline addressing support for family and carers |
| Self-Management and Psychosocial Factors | 1. Should clinical guidelines address self-management and psychosocial factors that may predict what patients may benefit from FES 2. No, the clinical guidelines should focus on FES as an intervention and not address self-management and/or psychosocial factors that might predict potential to benefit. 3. Yes, it would be valuable to include in a longer section of general information more detail about possible influences of self-management and psychosocial factors 4. Yes, there should be a clinical guideline on how to assess self-management and psychosocial factors that may influence ongoing use of FES and make decisions about provision 5. Yes, there should be a clinical guideline on how to support self-management when using FES |
| Awareness of FES and Sign Posting | 1. Should clinical guidelines address strategies to increase awareness of FES? 2. No, the clinical guidelines should not address strategies to increase awareness of FES. 3. Yes, it would be valuable to include in a longer section of general information more detail about strategies to increase awareness of FES with all stakeholders 4. Yes, it would be valuable to include in a longer section of general information more detail about strategies to increase awareness of FES specifically with appropriate referrers 5. Yes, there should be a clinical guideline relating to optimising strategies to increase awareness of FES with all stakeholders 6. Yes, there should be a clinical guideline relating to optimising strategies to increase awareness of FES specifically with appropriate referrers |
| Initial Referral for FES | 1. Should clinical guidelines address the optimal route for initial referral of people for FES? 2. No, the clinical guidelines should not address the optimal route for referral 3. Yes, there should be a clinical guideline indicating best practice for referral, with no differences for people with different conditions 4. Yes, there should be clinical guideline indicating best practice for referral with differences according to a person’s clinical condition or according to different service pathways 5. How should patients ideally be referred for FES? 6. Any allied health professional or medical consultant 7. G.P or community healthcare professional 8. Self-referral 9. Combination of a, b or c |
| Access to FES | 1. Should clinical guidelines address optimal routes to access and funding for FES? 2. No, the clinical guidelines should not address optimal routes to access and funding. 3. Yes, there should be a clinical guideline indicating best practice for optimal routes to access and funding, with no differences for people with different conditions 4. Yes, there should be clinical guideline indicating best practice for optimal routes to access and funding, with differences according to a person’s clinical condition or according to different service pathways |
| Assessment and Treatment with FES | 1. Should clinical guidelines address how patients are assessed and treated with FES? 2. No, the clinical guidelines should not address assessment and treatment 3. Yes, there should be a clinical guideline with best practice in relation to selection criteria and how these are assessed 4. Yes, there should be a clinical guideline with best practice in relation to the duration and intervals for assessment and treatment sessions 5. Yes, there should be a clinical guideline with best practice in relation to the provision of training / education for the person in how to independently use FES |
| Follow-up of patients with FES devices | 1. Should clinical guidelines address optimal processes for follow-up while people are using FES? 2. No, the clinical guidelines should not address follow-up 3. Yes, there should be a clinical guideline that recommends follow-up initially after three months, then after six months, and then at annual intervals while a person is using FES 4. Yes, there should be a clinical guideline that recommends follow-up but that does not specify the time intervals due to differences in service design and funding 5. Yes, there should be a clinical guideline that indicates a service will provide telephone and technical support as needed |
| Measuring Progress and Outcome Measures | 1. Should Clinical Guidelines include information about ongoing monitoring while using FES? 2. No, the clinical guidelines should not address ongoing monitoring as assessments are service-specific 3. Yes, there should be a clinical guideline that recommends specific, agreed objective and clinical outcome measures 4. Yes, there should be a clinical guideline that recommends monitoring of self-reported variables relating to the person’s quality of life and participation in life 5. There should be more research into variables that could be valuable for assessment in the future, such as physiological cost of walking, use of the FES device, and physical activity levels. |
| Clinical Training and Knowledge | 1. Should Clinical Guidelines address minimum training and development requirements to use FES? 2. No, the clinical guidelines should not address minimum training and development requirements to use FES 3. Yes, there should be a clinical guideline recommending a minimum of one day of initial training in FES 4. Yes, there should be a clinical guideline recommending a minimum of two days of initial training in FES 5. Yes, there should be a clinical guideline indicating that FES devices should not be sold to people who have not attended a device-specific training course 6. Yes, there should be a clinical guideline indicating a requirement for minimum numbers of years in practice as a physiotherapist, with expertise developed in neurological physiotherapy 7. Yes, there should be a clinical guideline indicating a requirement to maintain practice in providing FES for a minimum number of hours each month 8. Yes, there should be a clinical guideline indicating a requirement to engage in specific continuing professional development / updates relating to FES provision every year |
